# Evaluation of Antireflux Mucosectomy for Severe Gastroesophageal Reflux Disease: Medium-Term Results of a Pilot Study

**DOI:** 10.1155/2022/1606944

**Published:** 2022-02-21

**Authors:** Arthur Laquière, Felix Trottier-Tellier, Romina Urena-Campos, Pascal Lienne, Laurence Lecomte, Maria Katsogiannou, Guillaume Penaranda, Christian Boustière

**Affiliations:** ^1^Department of Gastroenterology, Saint Joseph Hospital, Marseille, France; ^2^Department of Clinical Research, Saint Joseph Hospital, Marseille, France; ^3^AlphaBio Laboratory, Biostatistics Department, F-13003 Marseille, France

## Abstract

**Background:**

Antireflux mucosectomy, a new endoscopic treatment for gastroesophageal reflux disease, consists of endoscopic mucosal resection at the esophagogastric junction. This study aim was to evaluate the medium-term efficacy of the antireflux mucosectomy technique for patients with severe gastroesophageal reflux disease symptoms (proton pump inhibitor treatment-dependent or proton pump inhibitor treatment-resistant gastroesophageal reflux disease).

**Methods:**

Between January 2017 and June 2018, 13 patients with severe gastroesophageal reflux disease without hiatal hernia, with positive pH reflux, were included in this monocentric prospective pilot study. The primary outcome was clinical success, defined by improvement evaluated by the Gastroesophageal Reflux Disease Health Related Quality of Life Questionnaire at 24 months. Secondary outcomes were technical success, decreased use of proton pump inhibitors, patient satisfaction, and adverse events.

**Results:**

Thirteen patients [females = 8 (62%)], mean age 59 (range, 54-68), were included. The antireflux mucosectomy procedure had technical success in all patients. At 24 months, for 11 patients, gastroesophageal reflux disease symptoms were significantly improved, and mean gastroesophageal reflux disease score decreased from 33 (range, 26-42) to 3 (range, 0-7) (p = 0.001). Ninety-one percent (*n* = 10) of patients had a lower proton pump inhibitor intake at 24 months. One patient had 3 endoscopic balloon dilatations for EGJ stenosis, two patients had melena ten days after procedure, and seven patients had thoracic or abdominal pain. Patient's satisfaction at 24 months was 81%.

**Conclusions:**

In patients with severe gastroesophageal reflux disease, despite occurrence of several short-term adverse events, antireflux mucosectomy seemed effective in improving gastroesophageal reflux disease symptoms at 24 months. This trial is registered with ClinicalTrials: NCT03357809.

## 1. Introduction

Proton pump inhibitors (PPIs) are the gold standard for medical treatment of gastroesophageal reflux disease (GERD) [1]. Long-term PPI therapy may increase the risks of lung and gastrointestinal infections as well as renal failure [2–4]. In addition, the financial burden of GERD is the highest among all chronic gastrointestinal disorders, with total costs up to $15 billion a year [5]. Furthermore, the role of PPI therapy in decreasing the risk of GERD progression to adenocarcinoma of Barrett's esophagus is unclear [6]. A recent study showed that surgical antireflux therapy might decrease this risk [7]. Laparoscopic fundoplication is generally recommended when symptoms are poorly controlled with PPIs [1]. This surgical treatment is considered to be the gold standard, with optimal control of reflux in the short to medium term [8, 9]. However, this intervention exposes the patient to potential complications of gastrointestinal laparoscopy, which are rare but multiple [10]. Moreover, long-term efficacy is not optimal [11, 12].

Many endoscopic techniques for treating GERD have been proposed to achieve nonsurgical control. These endoscopic techniques are aimed at approximating tissues at the esophagogastric junction (EGJ) for staple or suture. However, a low response rate has been demonstrated, and no endoscopic procedure has been widely accepted due to insufficient symptom control and cost of devices [13–18]. Inoue et al. showed that healing of EGJ mucosectomy may reduce GERD symptoms [19]. Healing of mucosectomy area retracts tissue causing restriction of EGJ. Most patients have had excellent control of GERD symptoms associated with normalization of pH-metry [19]. A few patients presented with dysphagia requiring two or three balloon dilatations to treat EGJ stenosis. After more than 10 years of follow-up, these patients remained asymptomatic, without requiring prescription of PPIs and without recurrence of Barrett's epithelium [9]. This study suggested that endoscopic mucosectomy may be an effective antireflux procedure, with the advantage of leaving no prosthesis in place. Only a few previous reports have evaluated this new endoscopic technique.

Up to date, no study evaluated the efficacy of this technique on patients with severe GERD symptoms, who are of specific interest. The risk-benefit ratio of antireflux mucosectomy (ARMS) procedure, for these patients with severe symptoms, is more favorable.

The aim of our study was to evaluate the medium-term efficacy of the ARMS technique for patients with severe GERD symptoms (PPI-treatment-dependent or PPI-treatment-resistant GERD).

## 2. Materials and Methods

### 2.1. Study Design and Population

Between January 2017 and June 2018, we conducted a monocentric prospective study where we included 13 patients with severe GERD without hiatal hernia, with positive pH reflux monitoring (defined as an acid exposure time (AET), % time with pH < 4%–>6%) [20]. Dependent GERD was defined as requiring long-term PPI therapy (>6 months), for symptom control [21]. Refractory GERD was defined in patients with persistent reflux symptoms despite PPI optimization for at least 8 weeks, in the presence of a pathological documented gastroesophageal reflux [22]. Exclusion criteria were as follows: age < 20 years, primary esophageal motility disorders, sliding hiatal hernia > 3 cm in gastroscopy, Hill grade IV flap valve, history of GERD surgery, history of GERD endoscopic treatment, ischemic heart disease, chronic kidney disease, cirrhosis, respiratory illness, substance abuse, past surgery of esophagus/stomach, Los Angeles Grade D esophagitis, Barrett's esophagus with dysplasia or reliefs, and pregnancy. Severity of GERD symptoms was assessed pre- and post-ARMS at (2, 6, and 24 months) by using the simplified DeMeester score and the GERD-Health Related Quality of Life (GERD-HRQL) questionnaire [23]. The Digitrapper™ reflux testing system (Sandhill Scientific, Highlands Ranch, CO, USA), which analyzes symptoms using a software (Reflux Reader 6.1), was used in all patients, pre-and post-ARMS (6 months). Gastroscopy was performed in all patients in order to evaluate the grade of esophagitis. During endoscopy, distance between diaphragmatic pinch and *Z* line (squamocolumnar junction) was measured on the endoscope relative to the incisors. The gastroesophageal flap valve was used to describe grade of hiatus hernia [19]. Mucosectomy was scheduled within a week depending on the former results. The study protocol did not include routine esophagogastroduodenoscopy (EGD) follow-up. If significant symptoms persisted postprocedure, an additional laparoscopic antireflux surgery was programmed. Complications were assessed from per-procedure up to 30 days post-procedure. All patients were called by the study nurse the next day after the procedure in order to assess for early complications. Patients either were seen during consultation at 1, 6, and 24 months or were called by the investigators. The severity of complications was graded according to the American Society for Gastrointestinal Endoscopy lexicon criteria [24]. This study was approved by the French ethical committee (No. ID RCB: 2016-A01591-50) and was registered in the http://ClinicalTrial.gov database (NCT03357809). All participants provided written informed consent.

### 2.2. Definitions and Outcomes

The primary outcome was clinical success, defined as 50% symptom improvement at 24 months, evaluated by the GERD-HRQL questionnaire. Secondary outcomes were technical success, improvement in simplified GERD score, pH monitoring, decreased use of PPI, and adverse effects and patient satisfaction.

The technical success was defined by completion of esophageal mucosectomy of 1 cm in height and of 1/2 circumference at the EGJ, associated with gastric mucosectomy of 2 cm in height in 2/3 circumference at EGJ. A simplified score was used to evaluate GERD symptoms (heartburn-regurgitation-dysphagia) severity, grading them from 0 to 3 (Table 1).

pH monitoring was carried out at baseline and 6 months after ARMS. Percentage of time with pH < 4 and number of long reflux episodes were compared at baseline and 6 months later. Use of PPIs was evaluated by score grading the uptake frequency (from never to everyday). Adverse effects were digestive hemorrhage, perforation, and abdominal or thoracic pain. Gastrointestinal hemorrhage was defined as the need for endoscopic hemostasis or transfusion. Digestive perforation was defined as need of endoscopic or surgical closure. Abdominal and thoracic pain was evaluated by a visual analog scale (VAS). Patient satisfaction was evaluated 24 months after ARMS by the following question: would you do the ARMS procedure again?

### 2.3. Endoscopic Procedure

ARMS procedures [15] were performed under general anesthesia with tracheal intubation. Patients were placed in dorsal decubitus position. A GIF-HQ190 upper endoscope (Olympus) with a 2.8 mm channel endoscope was used to examine the esophagus and stomach. For mucosectomy, an electrocautery generator system (VIO®3 ERBE Elektromedizin, Tubingen, Germany) was used.

EGJ mucosectomy was performed according to the same protocol that Inoue et al. previously described [19]. A saline solution with indigo carmine (2/100) and adrenaline (1/1000) was injected into the submucosa with a 25 G needle for mucosal lifting. Piecemeal mucosectomy of EGJ was performed in a crescentic fashion using Endocut Q, effect 2 (Cutting Interval 3, Cutting Duration 3) with Snare Captivator™ (Boston Scientific, CO USA). The mucosa was resected (vertical extent) about 1 cm in the esophagus and 2 cm in the stomach. Piecemeal mucosectomy of EGJ was complete when hemicircumference of mucosal stomach and esophagus was resected. Complete hemostasis of bleeding submucosal vessels was achieved. The submucosa was inspected for bleeding and deeper layer injury. Hemostasis was carried out using coagulation forceps (Coagrasper, FD-410LR, Olympus) with soft coagulation current (effect 5, 80 W).

### 2.4. Postendoscopy Follow-Up

Patients were kept nil per os for 24 h postprocedure, started on clear fluids on the next day, and were advised to start a soft diet before slowly transitioning to solid food as tolerated. Parenteral PPI was given on the day of procedure; it was switched to oral 40 mg PPI for 4 weeks after the procedure and was then stopped. Patients were ideally discharged on the second day after the procedure. Postendoscopy abdominal pain was treated with antalgics.

### 2.5. Statistical Analysis

Descriptive statistics are presented using median and interquartile range (IQR) or frequency and percentage according to the type of data. Primary endpoint was analyzed using ANOVA for repeated outcome; GERD score was compared among time points using the Tukey approach with multiplicity adjustment. All *p* values were considered significant at *α* − level = 0.05. Calculations were performed using SAS V9.4 software (SAS Institute Inc., Cary, North Carolina, USA).

## 3. Results

13 patients [females 8(62%)], with a median age of 59 ([IQR 54-68) years old were included. Patient baseline characteristics and PPI treatment are described in Tables 2 and 3.

One patient had a low dose of PPI (several times a month), because of an effective alginate therapy (3 alginates per day).

One patient was lost to follow-up at 3 months, and one patient had antireflux surgery at 6 months. Twenty-four months after ARMS, GERD symptoms were significantly improved for the 11 remaining patients, for whom median GERD-HRQL score decreased from 33 (IQR 26-42) to 3 (IQR 0-7) (*p* = 0.001) (Table 4).

**Figure 1:**
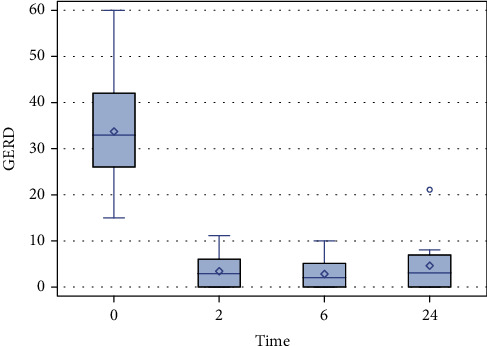
GERD-HRQL score distribution. Reflux symptoms progression reflected by GERD-HRQL scores at baseline, 2, 6, and 24 months post-antiflux mucosectomy.

ARMS efficacy was stable over time; 2 to 2 comparisons after multiple adjustments of 2-, 6-, and 24-month GERD-HRQL scores showed no difference (Figure 1).

Heartburn decreased for 11 patients (100%), and it had disappeared at 24 months after ARMS for 6 patients (55%). Regurgitation progressively decreased for 11 patients (100%) and disappeared for 10 (91%) of the patients after 24 months. For 11 patients (100%), dysphagia had disappeared at 24 months.

Ninety-one percent (*n* = 10) of patients had a PPI intake which dropped 24 months after ARMS, and treatment stopped for 6 (55%) of the patients. One patient maintained a daily uptake.

**Figure 2:**
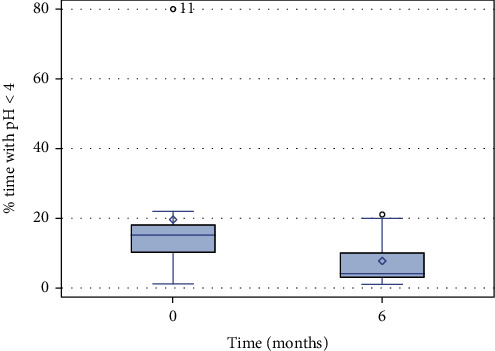
pH monitoring. Intragastric pH monitoring in patients at baseline and 6 months post-antiflux mucosectomy. Values represent rates of pH < 4.

However, 6 months after ARMS, there was no difference in results of pH monitoring compared to the baseline (Table 5, Figure 2).

We decided to suspend the inclusion in the study after 13 procedures due to the occurrence of several short-term adverse events. Intraoperative bleeding was observed in 13 (100%) patients, but endoscopic hemostasis was successfully achieved in all cases without need for additional blood transfusion. Two/13 patients had melena ten days after ARMS: one patient had pain after endoscopic hemostasis, for whom CT scan showed a small pneumoperitoneum and laparoscopy showed no gastrointestinal perforation. For the second patient, melena stopped spontaneously after two days. Seven/13 patients had immediate or postoperative thoracic or abdominal pain with the use of Class 1 or 2 analgesics for two or three days. For one patient, intermittent pain while swallowing persisted for 6 months. One/13 patient had 3 endoscopic balloon dilatations for EGJ stenosis. All the short-term adverse events have been resolved.

Despite these short-term adverse events, 24 months after ARMS, the overall patient satisfaction in reducing GERD symptoms was very good, 9/11 patients (81%) were willing to repeat ARMS if needed.

## 4. Discussion

ARMS has shown promising results in some studies and has the potential to become a significant treatment option for GERD, provided its efficacy and safety are demonstrated by large randomized studies.

The first study on ARMS results published by Inoue et al. showed ARMS clinical efficacy with improvement in DeMeester scores, with a 2-month follow-up [19]. Among patients who were able to discontinue PPI in the early postoperative period, the effect was maintained afterwards. Even with respect to subjective symptoms, treatment effect was observed several months after ARMS, and it was maintained even 1 year later. In addition, in their recent study Sumi et al. confirmed the 3-year beneficial effect [25]. In other recent studies, ARMS was also effective, but follow-up was short (a few months) and GERD symptoms were not severe [26–28]. In our study, all patients had severe dependent or refractory to PPI treatment GERD and post-ARMS follow-up lasted up to 24 months. Effectiveness of endoscopic treatment on GERD symptoms at medium-term with increment of the GERD-HRQL score was satisfactory, prolonged over time, and did not decrease.

In addition, 91% of patients reduced or stopped PPI treatment, and 84% patients agreed to repeat ARMS if necessary. ARMS was a technical success in 100% of the patients. However, pH monitoring criteria were not significantly different at 6 months after ARMS.

The lack of significant improvement in pH monitoring might be explained, in part, by an insufficient number of patients in the study. In addition, all selected patients had a severe GERD, suggesting that ARMS treatment was not effective enough for severe GERD. ARMS treatment seemed to be more appropriate for moderate GERD.

Regarding the characteristics of patients without symptoms improvement, we noticed that younger patients with more regurgitation had a lower response to ARMS treatment. These criteria need to be confirmed in further studies.

In addition, short-term tolerance was average with 64% (7/11) basal-thoracic pain occurring within 48 hours of endoscopic procedure, 18% (2/13) delayed upper gastrointestinal bleeding, and 9% (1/11) esophageal stenosis. Adverse events related to ARMS were frequently observed in previous studies, mainly digestive hemorrhage (hematemesis and melena) [27], digestive perforation [28], and abdominal pain.

Mucosal scarring is uncontrollable, making treatment effectiveness random, with some patients having no effectiveness and others having too much scarring that promotes stenosis. Furthermore, by leaving the mucosectomy scar open, delayed complications such as bleeding or perforation are increased. Some studies showed the impact of closing the mucosectomy area with a clip [29]. Mucosectomy of the EGJ associated with closure of resected mucosa may decrease risk of delayed bleeding, postendoscopy pain, and perforation. Closure with clips may also allow better calibration of tissue retraction. A recent study associated endoscopic mucosal resection with suture-plication with the Overstitch system [29]. The mean follow-up was 9 months, and there was significant improvement in their GERD-HRQL scores (*p* < 0.0001, 95% CI 19.3-25.3). Eight of 10 patients stopped their daily PPI dependence. However, no quantitative evaluation by pH monitoring was done, and follow-up was short. No digestive hemorrhage or digestive perforation occurred. Furthermore, patients did not experience any pain and were all safely discharged on the same day.

Another way to decrease adverse events consists in EGJ mucosal destruction; adverse events (perforation and hemorrhage) were in theory lesser than mucosectomy. One recent study of Inoue et al. showed effectiveness and safety of antireflux mucosal destruction using triangle-tip knife J in spray coagulation mode [30]. Another way to decrease the risk of EGJ stenosis consists in performing a modified area-mucosal ablation. Mucosectomy was done on both sides, like a butterfly [25]. More studies are needed in order to determine which method of EGJ treatment to use (ablation or destruction) and the mucosal area to treat.

In the selected population, 3 patients had short Barrett's esophagus (<3 cm) without dysplasia. However, the resection technique was standardized without specifically taking into account the presence of Barrett's esophagus and without evaluating their evolution after ARMS.

The major limitations of our study were the small number of patients and the lack of control group. However, patients formed, in term of GERD symptom severity, a homogeneous well-monitored group. In addition, the learning curve might have been another limiting factor because a single-experienced operator performed all procedures. Given the occurrence of short-term adverse events, leading us to suspend the inclusions in our study, the question of more appropriate inclusion criteria can be raised; ARMS might probably be more appropriate for patients with less severe GERD symptoms.

## 5. Conclusions

In conclusion, in this pilot study, ARMS of EGJ was a feasible and effective medium-term technique for GERD treatment with a relatively high morbidity rate. A less extensive mucosectomy with closure of the resected mucosal area should bring more consistent results and probably lower complication risks. Further larger and controlled studies are needed to evaluate this endoscopic technique in GERD treatment by improving inclusion criteria for a better efficacy/tolerability balance.

## Figures and Tables

**Table 1:** Simplified score used to evaluate GERD symptoms severity.

Severity of symptoms	Heartburn	Regurgitation	Dysphagia
0			
1			
2			
3			

0: none; 1: mild (could be ignored); 2: moderate (could not be ignored but did not affect lifestyle); 3: severe (affected lifestyle).

**Table 2:** Baseline characteristics of study population.

Characteristics	All patients (*n* = 13)
Age [years]	59 [54-68]
Gender	
Female	8 (62)
Male	5 (38)
BMI [kg × m^2^]	25.8 [25.0-28.1]
Duration of GERD symptoms [month]	62 [56-120]
Smoking	4 (31)
Esophagitis (LA classification)	
A	12 (92)
B	1 (8)
Hill's flap valve	
1	4 (30)
2	6 (46)
3	3 (23)
4	0 (0)
Major symptoms	
Heartburn DeMeester	
2	1 (8)
3	12 (92)
Regurgitation DeMeester	
1	2 (15)
2	6 (46)
3	5 (39)
Respiratory symptoms	1 (8)
Chest pain	2 (15)

Values are median (IQR) or numbers (%). BMI: body mass index; LA: Los Angeles.

**Table 3:** PPI treatment before ARMS.

PPI treatment frequency	
Every day	12 (92)
Several times a week	0
Several time a month	1 (8)
Several times a year	0
Never	0
PPI treatment dose (mg/day)	
20	5 (38)
40	8 (62)

Values are numbers (%).

**Table 4:** GERD-HRQL score progression during follow-up.

	Baseline GERD score	2-month GERD score	6-month GERD score	24-month GERD score
Overall score	33 [26-42]	3 [0-6]	2 [0-5]	3 [0-7]
Median difference between each time points and baseline [95% CI]	—	-30 [-39; -15]	-30 [-37; -15]	-28 [-35; -16]
*p* value	—	0.0010	0.0010	0.0010

Values are median [Q1Q3]. GERD: gastroesophageal reflux disease.

**Table 5:** pH monitoring.

pH monitoring	Baseline	6 months	Median difference [95% CI]	*p* value
Time pH < 4 [%]	15 [10-18]	4 [3-10]	-9 [-12; 3]	0.2266

Values are median [Q1Q3].

## Data Availability

The data used to support the findings of this study are available from the corresponding author upon request.
